# Shoulder pain: a hematologist’s perspective

**DOI:** 10.1007/s10195-016-0403-1

**Published:** 2016-04-13

**Authors:** Ankur Jain, Pankaj Malhotra, Subhash Varma

**Affiliations:** Department of Hematology, Nehru Hospital, PGIMER, 4th floor, Chandigarh, India

Sir,

Orthopedic surgeons commonly encounter cases of shoulder pain in their clinical practice, differential diagnosis of which includes, but is not limited to, septic arthritis. Usually perceived as ‘benign’, shoulder pain may be a harbinger of a serious underlying disorder including hematological malignancies. Jain et al. recently reported a case of chronic myeloid leukemia (CML) presenting as shoulder pain mimicking septic arthritis, and later diagnosed as myeloid sarcoma (MS) [1]. MS represents an extramedullary accumulation of immature cells of granulocytic series and occurs most commonly in the setting of acute myeloid leukemia (AML), where its incidence is 2–8 %, occurring after (50 %), prior to diagnosis (25 %) or concurrently (15–35 %) with AML and may rarely be a first site of AML relapse. Diagnosis requires fine needle aspiration (FNA) and immunohistochemistry (MS being positive for myeloperoxidase) [1]. Although bone and periosteum are amongst the commonest sites of MS, joint involvement is extremely rare and results from tumor invasion of the cortex and medulla resulting into a soft tissue mass [3]. Considering the shoulder as an important though rare site of MS, we reviewed all cases of MS in the English literature involving the shoulder. A brief review of all cases of shoulder MS, including their clinical/radiological findings, treatment and outcome is presented in Table 1 [1–6]. Amongst six cases of shoulder MS, CML was the commonest underlying etiology, and in one of them MS was an initial presentation of CML. Males in their fourth decade were most commonly affected. Pain and mass in the shoulder were the commonest presenting complaints. Examination could identify splenomegaly (two cases) and axillary lymphadenopathy (one case), and MRI could identify a soft tissue mass (five cases) with or without an associated lytic lesion. From an orthopedic view point, septic arthritis was the commonest primary diagnosis. All the cases of CML received tyrosine kinase inhibitors, and three of them also received an additional systemic chemo-radiotherapy. Prognosis of shoulder MS is guarded and long term survival is unreported. Though MS is uncommon in CML, myelodysplastic syndrome and other myeloproliferative neoplasms, our literature review identified CML as the leading diagnosis in cases of MS of the shoulder. Considering its rarity, no definite treatment guidelines are available. Although systemic chemotherapy followed by allogeneic stem cell transplantation clearly offers a survival advantage in cases of MS, lack of matched sibling/unrelated donors and financial costs are real concerns in developing countries like India, where combined chemo-radiotherapy holds promise as the best form of ‘palliation’ due to lack of its survival benefits and the poorer prognosis of such cases [1]. We conclude that, although septic arthritis is the commonest entity producing shoulder pain and swelling, presence of an associated lymphadenopathy, splenomegaly, soft tissue component with/without lytic lesion on MRI, peripheral leucocytosis with immature granulocytes/blasts, and absence of response to antibiotics should prompt an orthopedic surgeon to seek a hematology consultation maintaining a high index of suspicion for MS. Hematologists should henceforth realize the urgent need for FNA, and the importance of performing immunohistochemistry (IHC) in cases of septic arthritis with the above features being referred from the orthopedic side for timely and accurate diagnosis and treatment.

**Table 1:** Review of cases of shoulder myeloid sarcoma with clinical details, treatment given, and outcome

Study no.	Age	Sex	Author	Year	Clinical presentation	Time of presentation with shoulder sarcoma	Additional findings	Imaging features	Systemic involvement	Underlying diagnosis	Molecular/cytogenetic abnormalities	Treatment given	Outcome
1.	35	M	Levy et al. [2]	2014	Right posterior shoulder pain	Initial presentation at diagnosis	Firm mass at the back, axillary lymphadenopathy	Lytic lesion in inferior angle of scapula	2 % blasts in periphery (CML-CP)	CML	BCR-ABL1 (p210) rearrangement	Dasatinib followed by allogeneic SCT	Not reported
2.	38	M	Upadhyay et al. [3]	2014	Right shoulder pain	Diagnosed case of CML since 2009, on hydroxyurea for last 2 years	Swelling over anterolateral aspect of right proximal arm, splenomegaly	Soft tissue mass lesion involving proximal part of right humerus with cortical breaks in the humeral head and neck completely encasing and infiltrating it	CML-CP	CML	Not available	Systemic chemotherapy and RT	Died 6 months after diagnosis
3.	39	M	Cozzi et al. [4]	2004	Incidentally found to have myeloid sarcoma following a fracture after accident	Diagnosed as CML in 1989, received interferon, hydroxyurea and imatinib	Pain in left shoulder	Proximal humerus osteolytic lesion associated with extensive substitutive tissue	Bone marrow in CP	CML	Not available	Imatinib +dexamethasone + cytarabine followed by local RT	Died due to mycotic pulmonary infection
4.	40	M	Alkubaidan et al. [5]	2007	Painful swelling of left shoulder 1 year after Allo-SCT	Diagnosed as SDS in teenage years, on pancreatic enzyme supplementation, with history of MDS, and underwent Allo-SCT 1 year back	Avulsion fracture of greater tubercle	Soft tissue mass circumferentially engulfing the proximal humerus, the rotator cuff and the long head of biceps tendon	NA	Shwachman-Diamond syndrome (SDS)	NA	NA	NA
5.	13	M	Lincopan et al. [6]	2011	Mass in right shoulder	Initial presentation	Mass in inner thigh, rib cage, middle-posterior mediastinum	Soft issue masses in the sub-dermal region	Not present	Isolated MS	Trisomy 11	NA	NA
6	35	F	Jain et al. [1]	2016	Pain and swelling of left shoulder	Diagnosed as CML-CP in 2004 (on Imatinib 400 mg OD), progressed to AP in 2014 (imatinib 600 mg) and had left shoulder pain in 2015	Redness and induration of left shoulder, splenomegaly	MRI of the left shoulder showing an ill-defined heterogeneously enhancing lesion involving the muscles around the shoulder and infiltrating into clavicle	CML-CP (peripheral blood and Bone marrow)	CML	BCR-ABL (H396R mutation in kinase domain)	High dose imatinib, hydroxyurea, low dose cytarabine and local radiotherapy (RT)	NA

*CML* Chronic myeloid leukemia, *CP* chronic phase, *AP* accelerated phase, *SCT* stem cell transplantation, *NA* not available, *RT* radiotherapy
